# miR-451a levels rather than human papillomavirus vaccine administration is associated with the severity of murine experimental autoimmune encephalomyelitis

**DOI:** 10.1038/s41598-021-88842-z

**Published:** 2021-04-30

**Authors:** Momoka Nakashima, Kana Ishikawa, Aika Fugiwara, Kaiin Shu, Yoshimi Fukushima, Masaaki Okamoto, Hirotake Tsukamoto, Takahisa Kouwaki, Hiroyuki Oshiumi

**Affiliations:** Department of Immunology, Graduate School of Medical Sciences, Faculty of Life Sciences, Kumamoto University, 1-1-1 Honjo, Kumamoto, 860-8556 Japan

**Keywords:** Adjuvants, Human papilloma virus

## Abstract

Human papilloma virus (HPV) vaccine is currently the most effective prophylaxis to prevent cervical cancer. However, concerns regarding its potential severe adverse reactions have limited the vaccination rate. HPV vaccines have been determined to contain adjuvants which induce inflammation by the innate immune system and are crucial for triggering adaptive immunity. MicroRNA-451a (miR-451a) is located within circulating extracellular vesicles (EVs) and regulates the innate immune response. In this study, we examined the effect of HPV vaccines and EV miR-451a on murine experimental autoimmune encephalomyelitis (EAE), which is an autoimmune disorder that affects the central nervous system. Although HPV vaccine induced pro-inflammatory cytokine expression and macrophage cell death, it failed to exacerbate mouse EAE, whereas circulating EV miR-451a levels were associated with the severity of EAE. Since miR-451a knockout exhibited only marginal effect on the murine EAE clinical score, our data suggest that miR-451a levels reflect an unknown condition associated with EAE severity. Interestingly, excessive uptake of glucose increased EV miR-451a levels both in vitro and in vivo and also exacerbated mouse EAE. Therefore, environmental factors that increase EV miR-451a levels exacerbate the autoimmune disorder more than the HPV vaccine. These observations provide evidence for the safety of HPV vaccines.

## Introduction

Cervical cancer has been determined to be caused by an infection with certain types of human papillomavirus (HPV). HPV vaccines have been developed and are effective prophylaxes for prevention of cervical cancer^1^. HPV vaccines have been approved and administered worldwide, with a favorable safety profile. Although the safety of HPV vaccines has been confirmed in several clinical trials^2,3^; the vaccination rate has remained low in many countries, especially in Japan. This could be attributed, in part, to unconfirmed reports of adverse events following HPV vaccination reported in the Japanese media^4^. Acute disseminated encephalomyelitis (ADEM) is an autoimmune disorder of the central nervous system (CNS)^5^. It has been associated with several types of vaccines including influenza, hepatitis B, and measles vaccines^6^. Several cases of ADEM following HPV vaccination have been reported^7–9^. However, large cohort studies in Europe have demonstrated that HPV vaccines cause only marginal effects on autoimmune diseases including demyelinating diseases of the CNS^10,11^. Although clinical and large cohort studies have confirmed the safety of HPV vaccines, the identification of the molecular mechanisms underlying this safety profile is expected to ameliorate concern and increase the HPV vaccination rate.

Experimental autoimmune encephalomyelitis (EAE) is an autoimmune disorder affecting the CNS such as ADEM, and it represents an animal model of multiple sclerosis^12^. There was a report that HPV vaccines induce EAE-like symptoms in mice^13^; however, the paper was retracted because of an inappropriate experimental approach^14^. Cervarix, an HPV vaccine, contains the AS04 adjuvant, which includes monophosphoryl lipid A (MPLA) and aluminum adjuvants. Gardasil, another HPV vaccine, contains amorphous aluminum hydroxyphosphate sulfate^15^. Aluminum salts have been used for various types of vaccines^16^. Recent studies have shown that aluminum salts can activate the inflammasome and induce cell death. This leads to the release of host genomic DNA which functions as a damage-associated molecular pattern to activate TLR9^17^. MPLA is an agonist of TLR4^18^, and TLRs play an important role in inducing inflammation and the adaptive immune responses. However, there is no direct evidence that links these adjuvants to the autoimmune diseases.

Circulating extracellular vesicles (EVs) have been determined to regulate the onset and/or exacerbate EAE in mice^19,20^. EVs contain the exosomes and microvesicles that deliver functional proteins and RNAs, thereby regulating intercellular communications^21^. MicroRNAs (miRNAs) are known to attenuate target mRNAs and their translation^22^. EVs deliver miRNAs from donor to recipient cells and regulate the immune response^23^. Previously, we have shown that miR-451a levels in serum EVs are negatively correlated with serum pro-inflammatory cytokine levels or local inflammatory responses following influenza vaccination^24^. The miR-451a levels in serum EVs have been found to spontaneously fluctuate in healthy subjects^25^, and certain diseases or unknown environmental factors may affect EV miR-451a levels^26,27^. Although EV itself regulates the immune responses, it is also known that EV miRNAs can be used as biomarkers for several diseases, such as cancers and kidney diseases^28,29^. In this study, we compared the effect of an HPV vaccine on EAE with that of EV miR-451a and found that HPV vaccine exerts a minimal effect on murine EAE; however, EV miR-451a levels were significantly correlated with murine EAE clinical score. Our data indicate that EV miR-451a is a biomarker for the risk of the autoimmune disease and that some environmental factors that affect EV miR-451a levels have greater effects on the autoimmune disorder compared with the HPV vaccine.

## Results

### HPV vaccines trigger the innate immune response but do not exacerbate murine EAE

**Figure 1:**
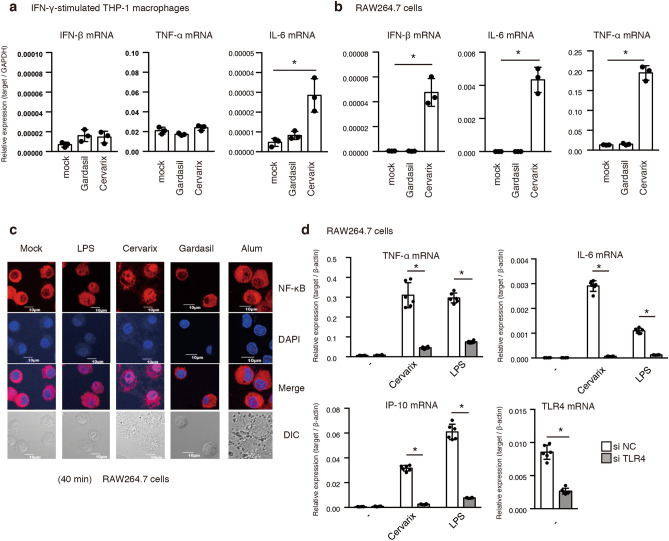
HPV vaccines induce pro-inflammatory cytokine expression. (**a**) THP-1 macrophages were treated with 10 ng/ml of IFN-γ for 24 h and then stimulated with 20 μl of mock solution, Gardasil, and Cervarix in a 24-well plate for 6 h. Cytokine expression was measured by RT-qPCR and normalized to GAPDH. Data represent the mean ± SD (n = 3, *p < 0.05, t-test). The data is a presentative of two independent experiments. (**b**) RAW264.7 cells were stimulated using 20 μl of mock solution, Gardasil, and Cervarix in a 24-well plate for 6 h. The expression of cytokines was determined by RT-qPCR and normalized to GAPDH. Data represent the mean ± SD (n = 3, *p < 0.05, t-test). The data is a presentative of two independent experiments. (**c**) RAW264.7 cells were stimulated with mock solution, 1 μg of LPS, 50 μl of Cervarix, 50 μl of Gardasil, and 200 μg of Alum in a 24-well plate for 40 min. Cells were then fixed and stained with anti-NF-κB Ab (red) and DAPI (blue). (**d**) 6 pmol of siRNA for the negative control or TLR4 was transfected into RAW264.7 cells for 48 h. Cells were then stimulated with PBS, 20 μl of Cervarix, or 1 μg of LPS in a 24-well plate for 6 h. The expression of cytokines and TLR4 was determined by RT-qPCR and normalized to that of β-actin. Data represent the mean ± SD (n = 6, *p < 0.05, t-test). The data is a representative of two independent experiments.

To examine pro-inflammatory cytokine expression in response to HPV vaccines, we measured the expression of IL-6, TNF-α, and IFN-β mRNA in macrophages following stimulation with two HPV vaccines. Treatment with Cervarix induced the expression of IL-6 in THP-1 macrophages and RAW264.7 cells (Fig. 1a and b). IFN-β and TNF-α expressions were also induced by Cervarix in RAW264.7 cells. In contrast, Gardasil slightly increased these cytokine levels (Fig.1a and b). Stimulation with Cervarix, as well as LPS, induced the nuclear localization of NF-κB, which is an essential transcription factor for pro-inflammatory cytokines (Fig. 1c). Since Cervarix contains MPLA, which is known to be a ligand of TLR4, we investigated whether an siRNA for TLR4 could reduce Cervarix-mediated cytokine expression. The siRNA for TLR4 and a negative control were transfected into the RAW264.7 macrophage cell line, and the cytokine expression was then measured by RT-qPCR. As expected, the expression of TNF-α, IL-6, and IP-10 mRNA was reduced by knockdown of TLR4 (Fig. 1d). We confirmed that the siRNA for TLR4 significantly reduced TLR4 mRNA levels (Fig. 1d). These data indicate that Cervarix can induce the cytokine expression through TLR4 in macrophage cell lines.

Considering that there are species-specific TLR4 responses^30,31^, the difference in the response between THP-1 and RAW264.7 cells might be caused by the difference in the species.

**Figure 2:**
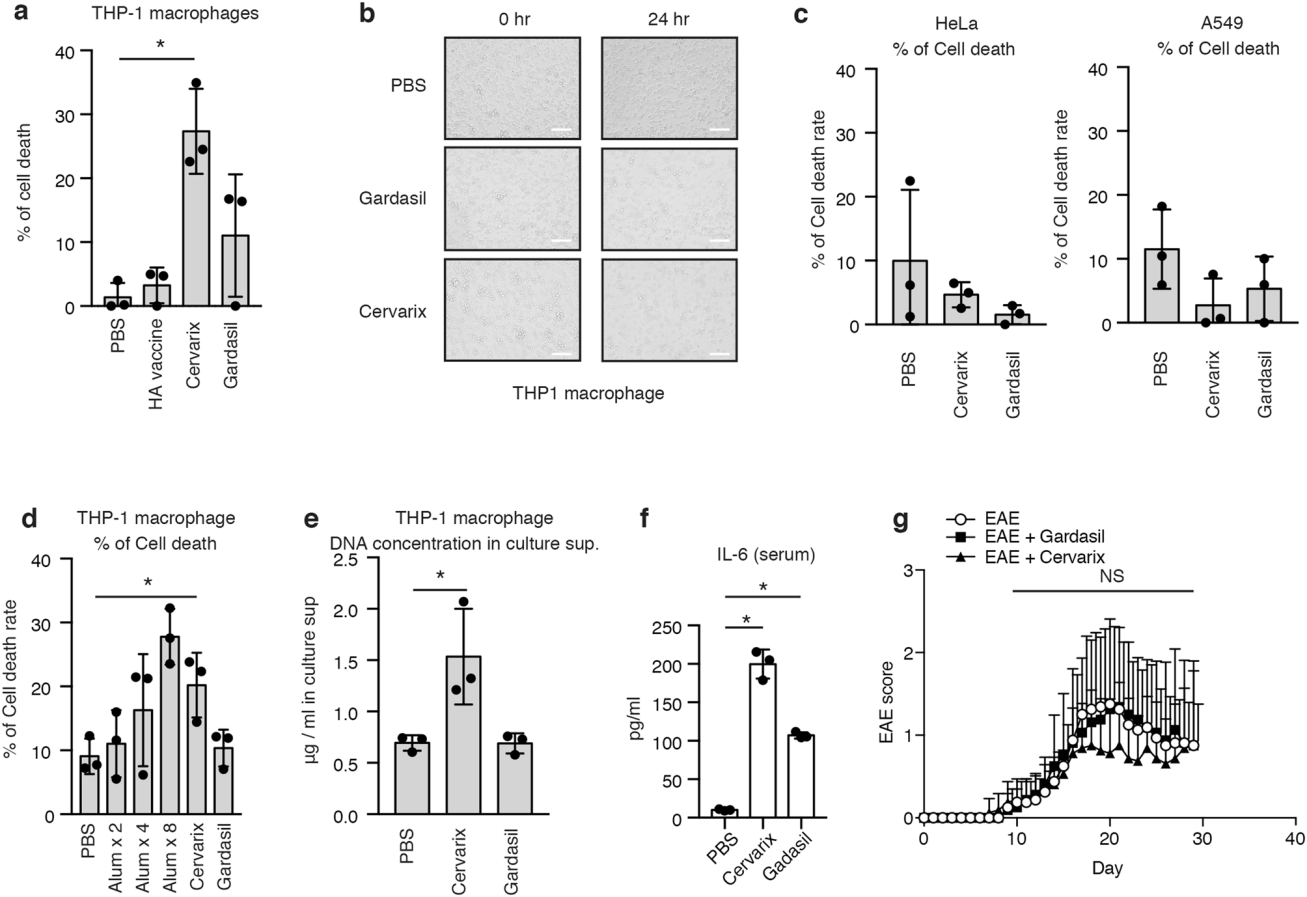
HPV vaccines fail to exacerbate murine EAE. (**a–c**) 4 μl of PBS, influenza HA vaccine, Cervarix, or Gardasil was added to the cell culture medium of THP-1 macrophages (**a,b**), HeLa cell (**c**), and A549 cells (**c**). 6 h after the addition of vaccine, the percentage of cell death rate was determined using a cell toxicity LDH assay kit (**a,c**). Data represent the mean ± SD (n = 3, *p < 0.05, t-test). The data is a presentative of two independent experiments. Cells were observed by confocal microscopy (**b**). The white bars represent 100 μm. (**d**) 4 μl of PBS; 0.1 μl (× 1), 0.2 μl (× 2), or 0.4 μl (× 4) of Imject Alum adjuvant; 4 μl of Cervarix; or 4 μl of Gardasil was added to the cell culture medium. 6 h after addition, the percentage of cell death rate was determined using a cell toxicity LDH assay kit. Data represent the mean ± SD (n = 3, *p < 0.05, t-test). The data is a presentative of two independent experiments. (**e**) 20 μl of PBS, Cervarix, or Gardasil was added to the cell culture medium of THP-1 macrophages in a 24-well plate for 6 h. Cell culture supernatants were collected. DNA were extracted and purified from cell culture supernatants using phenol, and DNA concentration were determined DNA by measuring absorbance. Data represent the mean ± SD (n = 3, *p < 0.05, t-test). The data is a presentative of two independent experiments. (**f**) Cervarix and Gardasil were intramuscularly injected into mice. 4 h later, sera were collected, and serum IL-6 levels were determined using ELISA. Data represent the mean ± SD (n = 3, *p < 0.05, t-test). The data is a presentative of two independent experiments. (**g**) Mice were immunized with MOG peptide to induce EAE. 100 μl of HPV vaccine or PBS was intramuscularly administered into mice at day 0. EAE clinical scores of the indicated groups (n = 16 mice per group) were evaluated for 30 days.

Both Gardasil and Cervarix contain aluminum adjuvants that induce cell death, leading to the release of cellular DNA, which in turn activates TLR9^17^. When Cervarix and Gardasil were added to cell culture medium of THP-1 macrophages, cell death was induced by Cervarix (Fig. 2a and b). Influenza HA vaccine, which does not contain aluminum adjuvant, did not induce cell death (Fig. 2a). In contrast, Cervarix and Gardasil treatments did not induce the cell death of HeLa or A549 epithelial cells (Fig. 2c). We confirmed that aluminum adjuvant could induce the cell death of THP-1 macrophages (Fig. 2d). We detected released DNA in the culture medium of THP-1 macrophages treated with Cervarix (Fig. 2e). Next, HPV vaccines were then administered into mice, and serum IL-6 levels were measured. Serum IL-6 levels were significantly increased following the administration of Cervarix or Gardasil (Fig. 2f). This in vivo data is consistent with our observation that HPV vaccines can induce pro-inflammatory cytokine expression and release cellular DNA in vitro.

Previously, Aratani S et al. reported that HPV vaccines induce murine EAE-like disease, suggesting that the HPV vaccine adjuvant is the cause of mouse EAE exacerbation^13^. Although HPV vaccines induce pro-inflammatory cytokine expression as described above, the administration of Gardasil and Cervarix did not affect murine EAE clinical scores (Fig. 2g and supplementary Fig. S1). Collectively, these data indicate the HPV vaccines do not affect the onset and exacerbation of the murine autoimmune disease.

### EV miR-451a levels are associated with the clinical score of murine EAE

**Figure 3:**
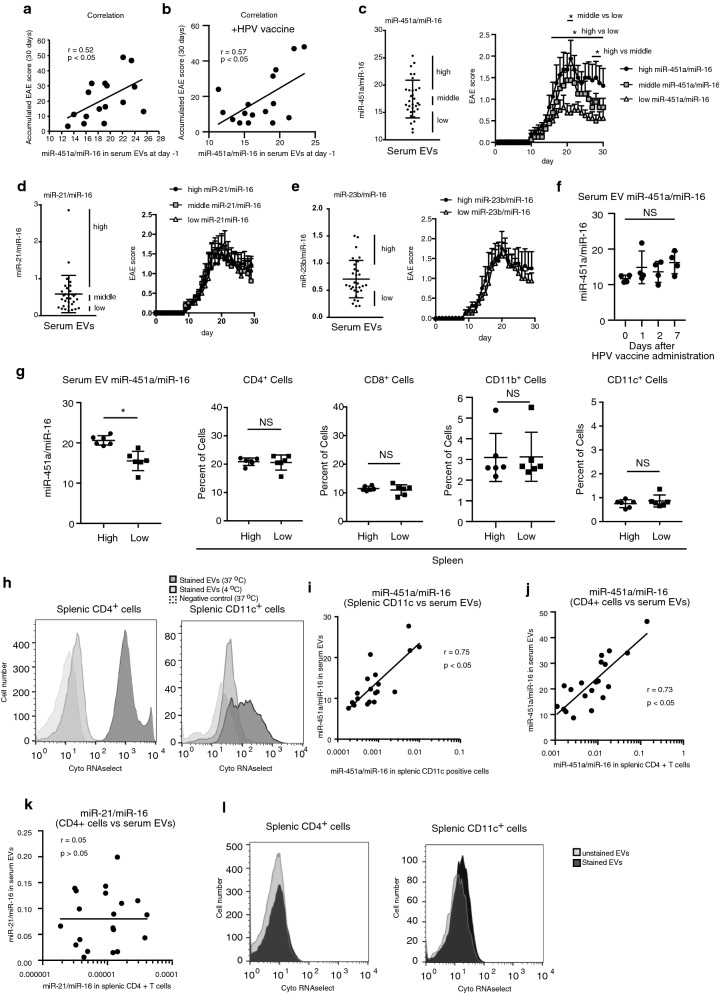
miR-451a levels in EVs are associated with the severity of murine EAE. (**a–e**) EVs were collected from sera of 16 (**a,b**) or 32 (**c–e**) mice (− 1 day), and miR-451a, miR-21, miR-23b, and miR-16 levels in EVs were measured by RT-qPCR. Mice were then immunized with MOG peptide to induce EAE. EAE clinical scores at day 30 were evaluated. Correlations between EAE clinical score (y-axis) and miR-451a/miR-16 ratio (x-axis) were determined (**a,b**). miR-451a/miR-16, miR-21/miR-16, and miR-23b/miR-16 ratios in serum EVs at − 1 day are shown in left panel (**c–e**). Mice were classified into three groups: high (top 8 mice), middle (8 mice from 13 to 20th), and low (bottom 8 mice). EAE clinical scores for each group were observed for 30 days (right panel, **c–e**). In panel **b**, Gardasil was administrated into mice before inducing EAE as described in Fig. 2g, and then miR-451a/miR-16 levels were compared with accumulated EAE scores. (*p < 0.05, 2-way ANOVA, n = 8 (each group)). (**f**) Cervarix was intramuscularly injected into mice, and then serum EVs were collected at indicated time points. EV miR-451a/miR-16 levels were determined by RT-qPCR (NS: p > 0.05, n = 4, t-test). (**g**) EVs were collected from the sera of 12 mice, and miR-451a and miR-16 levels in the collected EVs were measured by RT-qPCR. Splenocytes were collected from the same 12 mice, and the population of CD4 + , CD8 + , CD11b + , and CD11c + cells were determined by flow cytometry. The 12 mice were classified into high and low miR-451a/miR-16 expressing groups. Data represent the mean ± SD (n = 6, *p < 0.05, t-test). The data is a presentative of two independent experiments. (**h**) EVs were collected from mouse sera and stained with SYTO RNASelect dye. Stained EVs and unstained EVs (negative control) were incubated with splenic CD4 + or CD11c + cells at the indicated temperature for 6 h. Cells were then analyzed by flow cytometry. (**i–k**) EVs were collected from mouse sera (n = 20), and EV miR-451a/miR-16 (**g,h**) or mir-21/miR-16 (**i**) levels were measured by RT-qPCR. Splenocytes were then isolated from each mouse, and splenic CD11c- or CD4-positive cells were collected. Intracellular miR-451a/miR-16 levels were determined by RT-qPCR. The correlation between the EV miRNA ratio and intracellular miRNA ratio was determined. (**l**) Stained and unstained EVs were intravenously injected into mice. 24 h after injection, splenic CD4 + cells or CD11c + cells were isolated from mice, and then subjected to flow cytometry.

Next, we investigated the relation between EVs and murine EAE. We focused on EV miR-451a levels, because we have previously found that miR451a levels, which is normalized to miR-16 levels, are associated with the expression of inflammatory responses to the components of influenza A virus vaccines^24,25^. Interestingly, murine accumulated EAE scores during 30 days were significantly correlated with the levels of circulating miR-451a in EVs collected at day − 1 (Fig. 3a). This correlation was also observed even in the presence and absence of HPV vaccine administration (Fig. 3a and b). We separated mice into three groups (high, middle, and low) based on their EV miR-451a levels at 1 day before EAE induction (day − 1). The mice whose miR-451a levels were included in top and bottom thirds were classified into high and low groups, respectively. The high group mice exhibited significantly higher EAE scores compared with the middle and low groups (Fig. 3c). In contrast, miR-21 and miR-23b levels were not associated with EAE score (Fig. 3d and e). Cervarix administration did not affect the serum EV miR-451a levels (Fig. 3f), which is consistent to the observation that Cervarix did not affect the EAE scores as shown in Fig. 2g. There was no difference in the number of CD4 + , CD8 + , CD11b + , and CD11c + cells in the spleen between the high and low groups (Fig. 3g). Considering that high miR-451a levels, but not HPV vaccine administration, were associated with the murine EAE in a same experimental condition, our data suggest that the effect of HPV vaccine on mouse EAE is smaller than the effect of fluctuation of EV miR-451a levels of WT mice. Thus, we next investigated whether EV miR-451a itself regulates mouse immune response related to EAE or not.

Since EV miR-451a levels were correlated with EAE scores, we examined whether miR-451a was delivered from EVs to immune cells. EVs were then collected from mouse sera, and EV RNA was stained with a membrane-permeable dye, SYTO RNAselect, which emits green florescence. Stained EVs were then incubated with splenic CD4 + or CD11c + cells. As expected, stained EV RNAs were efficiently incorporated into splenic CD4 + cells and CD11c + cells (Fig. 3h). We have previously shown that EVs deliver miR-451a into recipient cells in vitro, thereby increasing intracellular miR-451a levels^24^.

Assuming EVs deliver miR-451a to recipient cells, it is expected that miR-451a levels in circulating EVs would be correlated with intracellular miR-451a levels in vivo. Serum EVs and splenocytes were collected, and miR-451a levels were measured using RT-qPCR. As expected, EV miR-451a levels in sera were associated with intracellular miR-451a levels in splenic CD11c + cells and CD4 + cells in vivo (Fig. 3i and j). In contrast, serum EV miR-21 levels were not correlated to intracellular miR-21 levels in the splenic CD4 + T cells (Fig. 3k). Because its expression levels in EVs were lower than miR-451a, it is possible that EVs could not deliver sufficient amounts of miR-21 to the splenocytes to change the intracellular miR-21 levels as discussed previously^24^. Although our data is consistent with the model that EVs delivers miRNAs into recipient cells in vivo, it is still possible that miR-451a levels reached an equilibrium between the EVs and the immune cells, because stained EVs injected into mice were only moderately incorporated into CD4 + T cells and CD11c + splenic cells in vivo (Fig. 3l).

**Figure 4:**
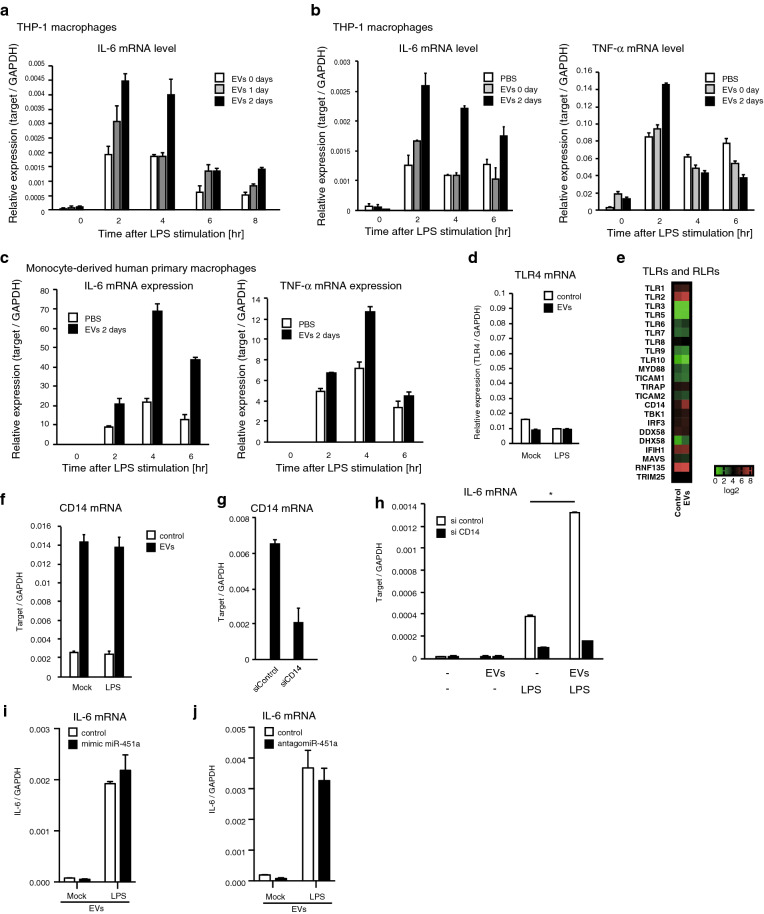
Serum EVs increase CD14 expression. (**a–c**) EVs were collected from human sera, and added to the cell culture medium of THP-1 macrophages (**a,b**) and monocyte-derived human primary macrophages (**c**) for 0, 1, or 2 days. THP-1 macrophages were stimulated with 0.1 mg/ml of LPS. Total RNA was extracted at indicated time points, and IL-6 and TNF-α mRNA levels were measured by RT-qPCR. (**d–f**) EVs were collected from human sera, and added to the cell culture medium of THP-1 macrophages for 2 days. THP-1 macrophages were then stimulated with 0.1 mg/ml of LPS for 2 h. Total RNA was extracted, and subjected to RT-qPCR (**d,f**) and a microarray analysis (**e**). (**g,h**) siRNAs for control and CD14 were transfected into THP-1 macrophages for 2 days. Simultaneously, cells were treated with EVs collected from human sera for 2 days. Cells were then stimulated with 0.1 mg/ml of LPS for 4 h, and then total RNA was extracted, and CD14 and IL-6 mRNA levels were measured by RT-qPCR. (**i,j**) miR-451a mimic RNA (**i**), antagomir-451a (**j**), and/or control RNAs were transfected into THP-1 macrophages for 2 days. Cells were then stimulated with 0.1 mg/ml of LPS for 2 h, and the expression of IL-6 mRNA were measured by RT-qPCR.

Next, we investigated whether EV alone could affect the immune response. EVs collected from human sera were added to cell culture medium of THP-1 macrophages, and their effect on cytokine expression was determined. Interestingly, LPS-induced IL-6 and TNF-α mRNA expression was augmented when cells were co-cultured with EVs for 2 days (Fig. 4a and b). EVs also increased the cytokine expression levels in monocyte-derived human primary macrophages (Fig. 4c). To determine the underlying mechanism, we performed microarray and RT-qPCR analyses. Since EVs increased the cytokine expression at 2 h after LPS stimulation, we compared EV-treated and untreated cells. We found that TLR expression levels were comparable between untreated and EV-treated macrophages (Fig. 4d and 4e); however, CD14 mRNA was significantly increased by treatment with EVs (Fig. 4e and f). Consistent with the increase of CD14, siRNA against CD14 reduced EV-mediated IL-6 expression (Fig. 4g and h). These data suggest that serum EVs have the potential to regulate the pro-inflammatory cytokine expression. To test the effect of miR-451a on EV-mediated enhancement of the cytokine expression, we utilized miR-451a mimic RNA and antagomir-451a. Unlike our expectation, miR-451a mimic RNA nor antagomir-451a failed to affect LPS-induced IL-6 expression in the presence of EVs (Fig. 4i and j). We do not exclude the possibility that miR-451a requires other miRNAs to affect the response to LPS.

**Figure 5:**
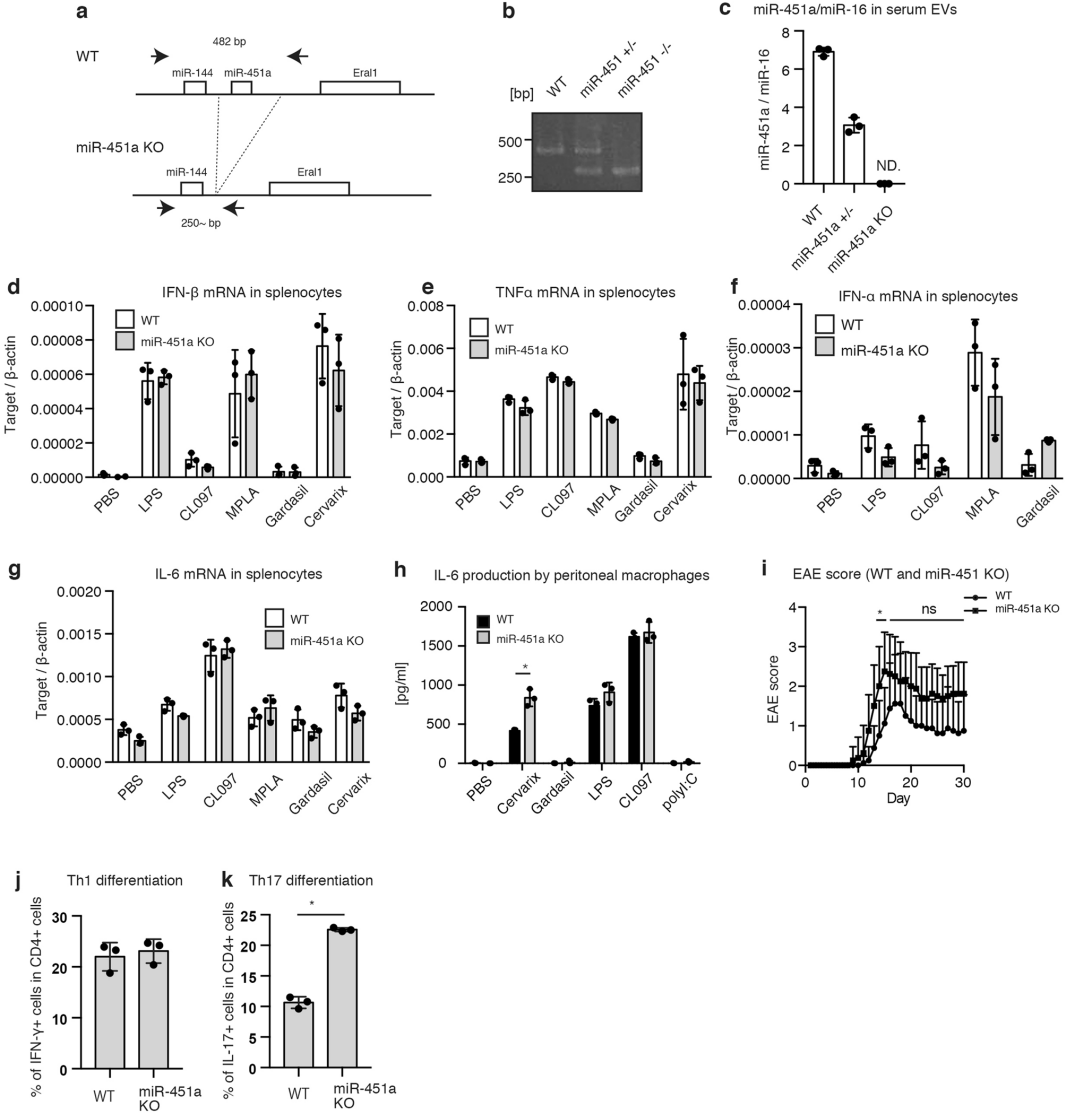
miR-451a KO fails to affect immune responses to adjuvants. (**a,b**) Schematic representation of WT and miR-451a KO mouse construction (**a**). Arrows in panel (**a**) represent the positions of the PCR primers used in (**b**). Genomic DNA was isolated from WT and miR-451a KO mice, and PCR was performed to confirm each genotype (**b**). (**c**) Serum EVs were collected from WT, miR-451a + /-, and miR-451a KO (n = 3). miR-451a and miR-16 levels in the collected EVs were measured by RT-qPCR. (**d–g**) Splenocytes were isolated from WT and miR-451a KO mice and stimulated with LPS (200 ng/ml), CL097 (5 μg/ml), MPLA (5 μg/ml), Gardasil (40 μl/ml), and Cervarix (40 μl/ml) in a 24-well plate for 2 h. Total RNA was extracted from cells, and the expression of the cytokines were measured by RT-qPCR and normalized to that of β-actin. Data represent the mean ± SD (n = 3, *p < 0.05, t-test). The data is a presentative of two independent experiments. (**h**) Peritoneal macrophages were isolated from WT and miR-451a KO mice, and then stimulated with LPS (200 ng/ml), CL097 (5 μg/ml), MPLA (5 μg/ml), Gardasil (40 μl/ml), and Cervarix (40 μl/ml) in a 96-well plate for 24 h. The IL-6 protein levels in culture media were determined by ELISA. (**i**) EAE was induced in WT and miR-451a KO mice, and EAE scores were evaluated for 30 days. (*p < 0.05, 2-way ANOVA, n = 8 (each group)). (**j,k**) Naïve CD4 T cells were isolated from the spleen of WT and miR-451a KO cells. Cells were then differentiated into Th1 (**j**) or Th17 cells (**k**) and were subjected to flow cytometry. IFN-γ^+^ and IL-17^+^ cells were counted as Th1 and Th17 cells.

To further assess the role of miR-451a in mouse EAE exacerbation, we performed miR-451a KO analysis. We confirmed that miR-451a knockout mice lacked the miR-451a gene, and serum EV miR-451a was not detectable in the knockout mice (Fig. 5a–c and supplementary Fig. S2). However, miR-451a knockout did not show any defect in the expression of IL-6, IFN-β, IFN-α, and TNF-α mRNA of splenocytes in response to TLR ligands, including LPS, CL097, and MPLA, as well as to Cervarix and Gardasil in the splenocytes (Fig. 5d–g). IL-6 productions from peritoneal macrophages of miR-451a knockout mice in response to LPS and CL097 were comparable to those of WT mice (Fig. 5h), whereas miR-451a KO mice moderately increased IL-6 production by macrophages stimulated with Cervarix (Fig. 5h). Unlike our expectation, miR-451a knockout did not ameliorate the murine EAE scores, and it rather increased the scores at a few time points (Fig. 5i). When naïve CD4 T cells were differentiated into Th1 or Th17, miR-451a knockout increased the efficiency of differentiation into Th17 but not Th1 cells (Fig. 5j, k, and supplementary Fig. S3). It is notable that Th17 is reported to exacerbate mouse EAE^32^. Since miR-451a knockout did not ameliorate the mouse EAE clinical symptoms, these data weakened the possibility that the increased level of serum EV miR451a in WT mice exacerbated mouse EAE.

Collectively, based on our in vivo and in vitro data, we prefer the interpretation that the difference of miR-451a levels reflects some unknown physical or environmental conditions that affect mouse EAE scores (see discussion).

### High glucose conditions lead to an increase of miR-451a levels and exacerbation of murine EAE

**Figure 6:**
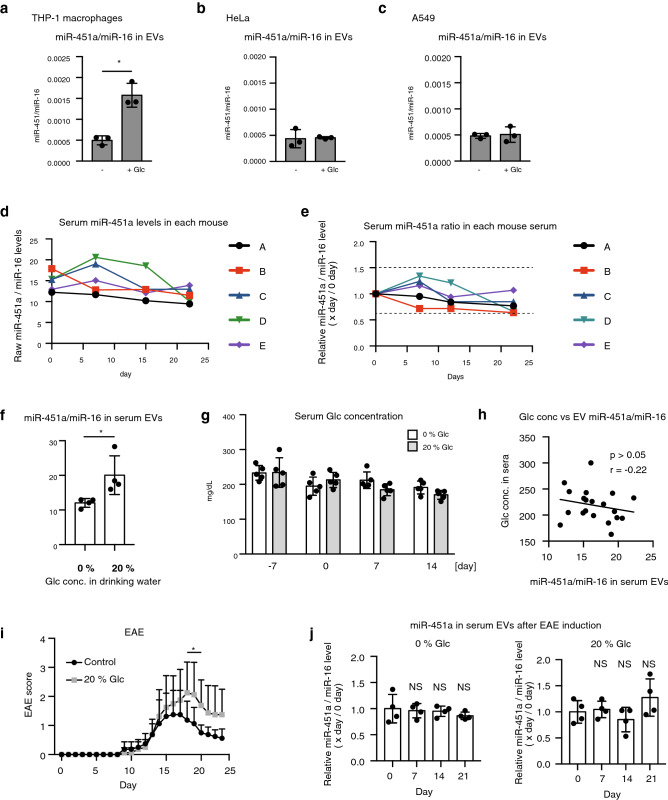
Excessive glucose uptake increases miR-451a/miR-16 levels and exacerbates murine EAE. (**a–c**) THP1 macrophages (**a**), HeLa (**b**), and A549 (**c**) cells were cultured in RPMI medium with or without glucose (4.5 g/L) for 24 h. EVs were collected from culture supernatants, and miR-451a and miR-16 levels were determined by RT-qPCR. (n = 3, The data represents the mean ± SD, *p < 0.05). (**d,e**) Mouse sera were collected for 24 days, and EVs were isolated from collected sera. miR-451a/miR-16 levels in serum EVs were determined by RT-qPCR. The y-axis represents miR-451a/miR-16 levels (**d**) or relative mir-451a/miR-16 levels (**e**). Relative miR-451a/miR-16 levels were calculated by dividing each miR-451a/miR-16 level by those at day 0. (**f–h**) Mice were treated with 20% glucose in drinking water for 2 weeks. miR-451a/miR-16 levels in serum EVs were measured by RT-qPCR. Serum glucose concentrations were measured (**g**) and compared with miR-451a/miR-16 levels in serum EVs (**h**). Data represent the mean ± SD (n = 6, *p < 0.05, t-test). The data is a presentative of two independent experiments. (**i**) Mice were treated with 20% glucose in drinking water for 2 weeks, and then EAE was induced to control and 20% glucose treated mice. EAE scores were observed for 25 days. (NS > 0.05, 2-way ANOVA, n = 8 (each group)). (**j**) Mice were treated with 20% glucose in drinking water for 2 weeks, and then EAE was induced to control and 20% glucose treated mice. EVs were collected from mouse sear at indicated time points, and miR-451a/miR-16 levels were determined by RT-qPCR (n = 4, *p < 0.05, t-test).

To test the hypothesis that some environmental factors that increase the miR-451a levels exacerbate the autoimmune disease, we focused on the effect of excessive glucose uptakes, as previous studies have shown that high glucose levels increase miR-451a expression^33^. When THP-1 cells were cultured in a glucose-containing medium, miR-451a levels in released EVs were increased compared with the controls (Fig. 6a). However, an increase of miR-451a in EVs was not observed when HeLa or A549 cells were cultured with glucose (Fig. 6b and c), suggesting that glucose in the culture medium increased EV miR-451a levels in a cell type-specific manner.

Next, we investigated the spontaneous changes in serum EV miR-451a levels in mice. Serum EV miR-451a levels gradually changed, but it did not surpass twofold over a 1-month period (Fig. 6d and e). When mice were bred with drinking water containing 20% glucose, serum EV miR-451a levels were significantly increased compared with controls (Fig. 6f). However, serum EV miR-451a levels did not reflect the serum glucose concentration (Fig. 6g and h), suggesting that miR-451a levels were associated with stress induced by high glucose condition. Interestingly, the high glucose condition significantly exacerbated murine EAE scores (Fig. 6i), which is consistent with a recent report^34^. We found that induction of EAE did not increase serum EV miR-451a in the absence of 20% glucose treatment (Fig. 6j), which is consistent to the notion that increased miR-451a level itself does not affect the onset or exacerbation of mouse EAE. Because mice uptook 20% glucose water more than 0% glucose water (supplementary Fig. S4), it is still possible that excessive uptakes of water affected mouse EAE scores. Collectively, our data indicate that environmental conditions results in a higher effect on murine EAE compared with HPV vaccines.

## Discussion

Administration of Gardasil and Cervarix to young adults has been approved and administrated into young women worldwide; however, the vaccination rate is not sufficiently high in many countries including Japan^4^. Adjuvants were once suspected to be harmful to the host. HPV vaccines induce pro-inflammatory cytokine expression, resulting in local inflammatory responses that are known to cause pain, redness, and swelling^35–37^, which are well-known adverse reactions of vaccination. In our study, however, HPV vaccines did not cause any effects on murine EAE, which suggest that local adverse reactions do not lead to more severe adverse reactions, such as autoimmune diseases in the CNS. Serum EV miR-451a levels were associated with EAE scores, and high glucose uptake increased both serum EV miR-451a levels and murine EAE scores. These observations indicate that the effect of HPV vaccines on the autoimmune disease in the CNS is much lower compared with environmental factors, such as high glucose uptake. Moreover, the spontaneous increase of miR-451a levels in serum EVs was associated with the severity of the murine EAE clinical symptoms. Therefore, the effect of HPV vaccine administration on autoimmune diseases is much less compared with the spontaneous increase of miR-451a levels. These observations provide additional evidence for the safety of HPV vaccines and should alleviate any concerns about vaccination.

EVs are released from various types of cells and include miRNAs. An accumulating body of evidence has shown that EVs regulate pro-inflammatory cytokine expression in the innate immune response. Our genetic data suggested that miR-451a knockout did not reduce EAE clinical scores but moderately exacerbated the clinical symptoms. We also found that miR-451a promoted the differentiation of naïve T cells into Th17 in in vitro experiments. Since Th17 promotes mouse EAE, increased EAE scores in miR-451a knockout mice might be caused by increased Th17 differentiation. miR-451a knockout also affected the cytokine expression in some experimental conditions. It should be noted that miR-451a levels in serum EVs were very high (over 1 × 10^9^ copies/ml in human sera)^24^, and thus it is expected that knockout of the miR-451a gene dramatically decreased miR-451a levels in circulating EVs. Considering that those dramatical decrease only moderately affected mouse EAE, the difference in miR-451a levels in circulating EVs in WT mice could not affect mouse EAE. Thus, we prefer the interpretation that high miR-451a levels are not a cause, but an effect of an unknown physical and/or environmental conditions in which autoimmune responses are augmented. MiR-451a levels are known to be increased during high glucose conditions^33^. Further studies are required to assess the role of miR-451a in circulating EVs on mouse EAE. Interestingly, our data showed that high glucose treatment increased not only miR-451a levels, but also murine EAE scores. These observations support the model that miR-451a levels reflect physical and/or environmental conditions that result in autoimmune diseases. The numbers of CD4^+^, CD8^+^, and CD11b^+^ cells were not different between miR-451a high and low groups, the physical condition might affect immune cell functions.

Our previous study has shown that miR-451a levels in circulating EVs gradually fluctuate, and the levels in healthy subjects may vary over 30-fold. In addition, several diseases are known to affect miR-451a levels in the blood. For instance, rheumatoid arthritis is associated with increased miR-451a levels^38^, and increased miR-451a levels were also observed in the sera of type 2 diabetes patients^39^. These diseases are known to be related to immune disorders. Thus, it is expected that miR-451a is an important biomarker for abnormal regulation of the immune system.

## Materials and methods

### Animals, cells, and reagents

HeLa cells were kindly gifted from Prof. Seya T (Hokkaido University) and were cultured in Eagle’s MEM with 10% fetal calf serum (FCS). RAW264.7 cells were cultured in D-MEM containing low glucose and 10% FCS. A549 cells (provided by T. Seya at Hokkaido University) were cultured in DMEM (high glucose) with 10% FCS. THP1 cells were purchased from the JCR cell bank. THP-1cells were cultured in RPMI-1640 containing 5% FCS. THP-1 cells were differentiated into macrophages with the addition of PMA (60 ng/ml) for over 16 h, as has been described previously^40,41^. Human monocyte-derived macrophages were generated from CD14 + monocytes isolated from human blood and cultured with human GM-CSF for 6 days^42^. Mouse splenocytes were prepared from the spleen and suspended in RPMI-1640 medium containing 10% FCS and 55 μM of 2-mercaptoethanol. Isolated cells were centrifuged at 1500 rpm for 5 min, and the supernatants were then discarded. Red blood cells were excluded using red blood cell lysis buffer. The resulting cells were diluted 10 times and centrifuged at 1500 rpm for 10 min. Cell pellets were resuspended in RPMI-1640 medium containing 10% FCS and 55 μM of 2-mercaptoethanol and cultured. Small interfering RNAs for negative control and TLR4 were purchased from Thermo Fisher Scientific. Influenza HA vaccine, Gardasil, and Cervarix were purchased from Daiichi Sankyo, Merck & Co., and GSK, respectively. Human EVs were collected form healthy human sera. This study was approved by the ethics committee of the Faculty of Life Sciences at Kumamoto University (RINRI 1524). All participants provided written informed consent, and all experiments have been conducted according to the principles expressed in the Declaration of Helsinki. Mimic miR-451a, antagomir-451a, and control RNAs were purchased from ThermoFisher Scientific.

### Mice

C57BL/6 mice were purchased from Kyudo. MiR-451a KO mice (MiR451^tm1.2Doca^) were purchased from the European Molecular Biology Laboratory. For miR-451a KO mice, the miR-451a gene was deleted within the miR-144/miR-451 cluster was deleted, leaving the expression of miR-144 intact^43^. Mice were back-crossed for five generations to C57BL/6 mice. The deletion of miR-451a was confirmed by PCR using the following primers: forward, 5′-CTG ACT GAC CCT GAG GCA AT-3′; reverser, 5′-CCA GCC TCG GAT GCT AAT AA-3’.

### Preparation of EVs

EVs were collected from human sera of healthy subjects and cell culture supernatants using a total exosome isolation reagent, from cell culture media and from serum (Thermo Fisher Scientific), according to the manufacturer’s instructions. To collect EVs from cell culture supernatants, cells were cultured in the appropriate medium without FCS for 2 days. To measure miRNA levels, total RNA was extracted from collected EVs and were then isolated using TRIzol reagent (Thermo Fisher Scientific). Complementary DNA was synthesized by reverse transcription using the miR-X-miRNA First-Strand Synthesis Kit (Clontech). RNAs within EVs were stained with SYTO RNAselect dye (Thermo Fisher Scientific).

### qPCR

To measure relative mRNA levels, total RNA was isolated using TRIzol reagent (Thermo Fisher Scientific), and cDNA was prepared using the High-Capacity cDNA Reverse Transcription Kit with an RNase Inhibitor (Thermo Fisher Scientific). Real time PCR was performed using a SYBR Green Real-Time PCR Master Mix and a Step One Real-Time PCR instrument (Thermo Fisher Scientific).

### Cell death

The cytotoxicity of the HPV vaccines and adjuvants were determined using the Cytotoxicity LDH Assay Kit-WST (Dojindo Molecular Technologies Inc.), according to the manufacturer’s instructions. Cells were seeded into a 96-well plate and incubated for 24 h. HPV vaccines or adjuvants were then added to the cell culture medium at 37 °C for 30 min. Then, 0.1 ml of culture medium from each sample was transferred into a new plate, and 0.1 ml of working solution was added to each well, and the plates were incubated for 30 min. Stop solution (0.05 ml) was added to each well to stop reaction, and the LDH activities were calculated by measuring the absorbance at 490 nm using a plate reader.

### Microarray

Exosome free serum (Exosome-depleted FBS) was purchased from System Biosciences. THP-1 macrophages were seeded into 24-well plates and cultured in RPMI-1640 with exosome-free serum supplemented with or without EVs collected from 100 μl of human serum and then stimulated with mock solution or LPS for 2 h. Total RNA was extracted from each sample and subjected to microarray analysis. Microarray data were then deposited into the DDBJ Sequence Read Archive (Accession No. GSE127714). Heatmap was drawn with Prism 7 Mac OS X ver. 7.0a.

### Confocal microscopy

Cells were cultured on a microscope cover glass slides (Matsunami) in a 24-well plate for 24 h. Cells were then fixed with 3% formaldehyde in phosphate-buffered saline (PBS) for 30 min at room temperature, and then permeabilized with 0.2% of Triton X-100 for 15 min. The cells were then blocked with 1% of BSA in PBS were used for blocking of cells and incubated for 10 min, and then cells were labeled with primary antibodies for 1 h. Cells were washed four times, and incubated with secondary antibodies for 30 min. Then, cells were washed again four times, and cover glasses were mounted onto the glass slides with ProLong Gold (Invitrogen) containing DAPI.

### EAE

C57BL/6JJcl female mice were purchased from CLEA Japan. The mice were 8 weeks-old at the beginning of the study. On day − 1, sera were collected form mice, and EVs were collected from sera. MiRNA levels in serum EVs were then determined by RT-qPCR. EAE was induced on day 1 with a subcutaneous injection of 200 µg of myelin oligodendrocyte glycoprotein (MOG_35–55_) peptide (BEX, Japan) emulsified with complete Freund's adjuvant (WAKO, Japan) containing 500 µg of heat-killed Mycobacterium tuberculosis H37 Ra. This was followed by an intraperitoneal injection of 500 ng of pertussis toxin (CALBIOCHEM) immediately and 48 h later. HPV vaccine was provided by MSD. On day 0, mice were intramuscularly injected with 100 μl of PBS or HPV vaccine. After EAE induction, the mice were monitored daily using the following score: 0, no symptom; 0.5, tip of tail is limp; 1, limp tail; 1.5, limp tail and hind leg inhibition; 2, limp tail and weakness of hind legs; 2.5, limp tail and dragging of hind legs; 3, complete hind limb paralysis; and 3.5, in addition to the symptoms score 3, hind legs are together on one side of the body. Since EAE scores decreased gradually, we determined the accumulated EAE clinical score that is the sum of EAE scores every day during 30 days.

### T cell differentiation

CD62L^hi^ naïve CD4^+^ T cells were isolated by naïve CD4^+^ T-cell isolation kit (Miltenyi Biotec) from pooled splenocytes of WT or miR451a KO mice, and then the naïve T cells (5 × 10^5^ cells ml^−1^) were stimulated with plate-bound anti-CD3 and anti-CD28 Abs (BD Biosciences) in the presence of IL-12 (8 ng ml^−1^; Wako) for Th1 differentiation, or of Th17 differentiation reagents (contained in Th17 Cell Differentiation Kit from R&D Systems) for 5 days. For intracellular cytokine staining, in vitro generated effector CD4^+^ T cells were re-stimulated with phorbol-12-Myristate-13-acetate (PMA) and ionomycin (both from Sigma) in the presence of Breferdin A (Sigma) for 5 h and then were fixed and permeabilized using BD Cytofix/Cytoperm Buffer (BD Biosciences). Those fixed cells were stained with Alexa Fluor 488-conjugated anti-IFN-γ (XMG1.2: Biolegend), and PE-conjugated anti-IL-17A Ab (eBio17B7: eBioscience). Immunofluorescent images and the data were analyzed using FACSCalibur (BD Biosciences) and FlowJo software (Tree Star), respectively.

### Statistical analysis

Statistical analyses were performed using Prism 7 software (GraphPad Software, Inc.) or MS Excel (Microsoft). Two-way ANOVA followed by multiple comparisons test was used to determine the statistical significance of the difference between two groups at different time points in mouse EAE experiments. Student t-test was used to investigate the statistical difference between the two data. P values lower than 0.05 were considered to be statistically significant. All graphs were drawn using GraphPad Prism Mac OS X ver. 7.0a.

### Ethics declarations

All the mice were housed in a pressure-controlled room at the Center for Animal Resources and Development, Kumamoto University, and all the experimental protocols were approved by the Institutional Animal Committee of Kumamoto University, and all the experimental methods were performed in accordance with accepted guidelines of the Institutional Animal Committee of Kumamoto University (G29-201). All studies were carried out in compliance with the ARRIVE guidelines.

## Supplementary Information

Supplementary Information

## Data Availability

Microarray data was deposited into the DDBJ Sequence Read Archive (DRA) with accession number GSE127714. All data generated or analyzed during this study are included in the publication article.
