# Supplementary material for: miR-451a levels rather than human papillomavirus vaccine administration is associated with the severity of murine experimental autoimmune encephalomyelitis
Source: Sci Rep. 2021 Apr 30;11:9369. doi: 10.1038/s41598-021-88842-z (PMC8087664; doi:10.1038/s41598-021-88842-z)
Supplement: Supplementary file 1 — Supplementary Information [file 41598_2021_88842_MOESM1_ESM.pdf]

Supplementary information

miR-451a levels rather than human papillomavirus vaccine administration is associated with the severity of murine experimental autoimmune encephalomyelitis

Momoka Nakashima, Kana Ishikawa, Aika Fugiwara, Kaiin Shu, Yoshimi Fukushima, Masaaki Okamoto, Hirotake Tsukamoto, Takahisa Kouwaki, *Hiroyuki Oshiumi

Department of Immunology, Graduate School of Medical Sciences, Faculty of Life Sciences, Kumamoto University, 1-1-1, Honjo, Kumamoto 860-8556, Japan

*To whom corresponding should be addressed: Hiroyuki Oshiumi (Department of Immunology, Graduate School of Medical Sciences, Faculty of Life Sciences, Kumamoto University, 1-1-1 Honjo Kumamoto 860-8556, Japan; oshiumi@kumamoto-u.ac.jp; Tel. +81-96-373-5134; Fax. +81-96-373-5138)

Supplemental Figure S1. Accumulated EAE scores of mice administrated with or without Gardasil or Cervarix
Supplementary Figure S2. Whole gel image of Fig. 5b
Supplemental Figure S3. Differentiation of naïve T cells into Th1 and Th17
Supplemental Figure S4. Intakes of water with or without Glc

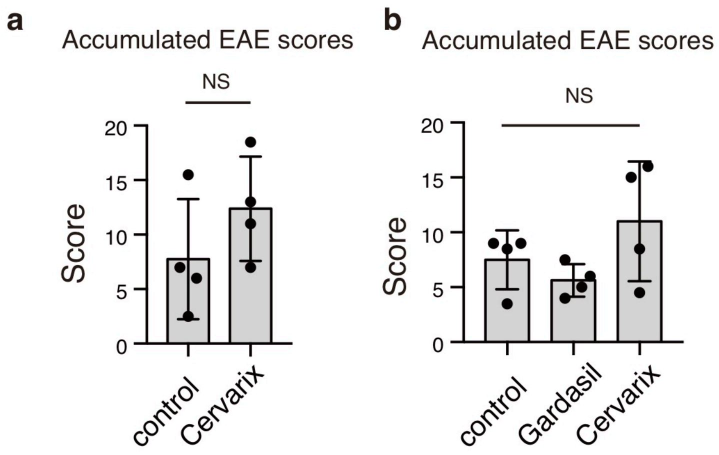

Supplementary Figure S1. Accumulated EAE scores of mice administrated with or without Gardasil or Cervarix

(a, b) WT mice were administrated with PBS, Cervarix (a, b), or Gardasil (b) 1 week before the induction of EAE. Accumulated EAE scores of the two groups were calculated (n = 4, t-test)

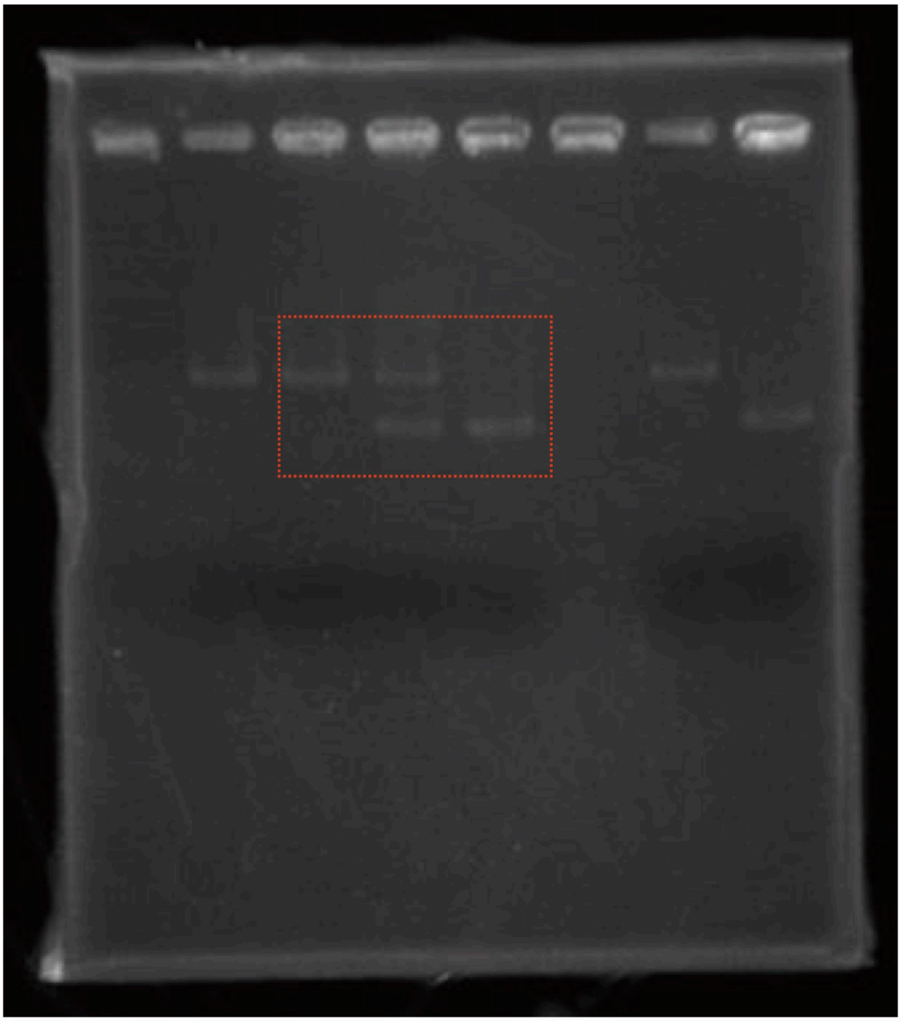

Supplementary Figure S2. Whole gel image of Fig. 5b

The region within the red rectangle is the image of Fig 5b.

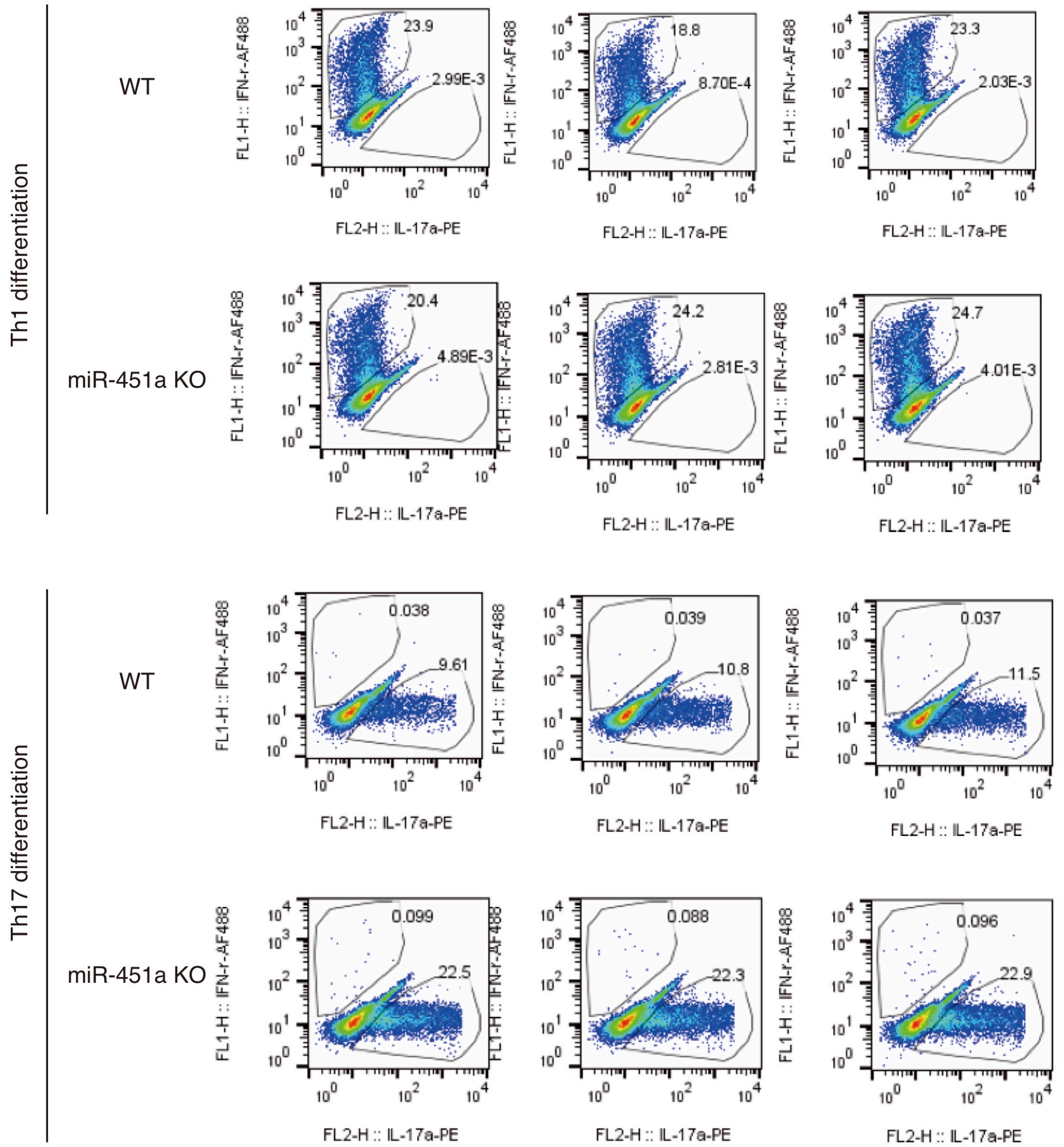

Supplemental Figure S3. Differentiation of naïve T cells into Th1 and Th17

Naïve CD4 T cells were isolated from the spleen of WT and miR-451a, and were differentiated into Th1 or Th17 cells with IL-12 (Th1) or Th17 differentiation kit (Th17) (R&D systems), according to the manufacture's procedure.

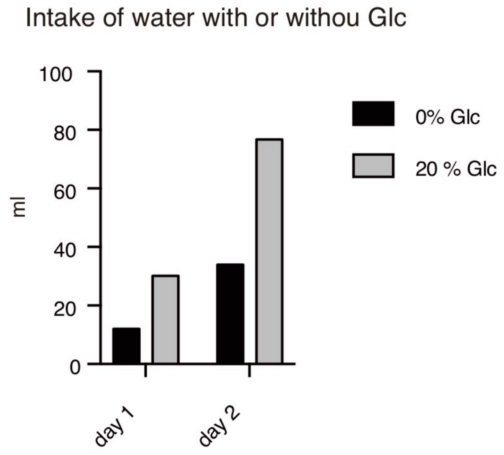

Supplemental Figure S4. Intakes of water with or without Glc

4 mice were put in a cage and bred with water with or without 20 % Glc. The amounts of intakes of drinking water of each cage were monitored for 2 days. The data is a representative of two independent experiments.
